# Biliary tree traumatic neuroma following laparoscopic cholecystectomy: A case report and literature review

**DOI:** 10.3892/mi.2023.97

**Published:** 2023-07-07

**Authors:** Hemn H. Kaka Ali, Dana T. Gharib, Marwan N. Hassan, Ari M. Abdullah, Deari A. Ismaeil, Omar H. Ghalib Hawramy, Dlshad H. Ahmed, Dilan S. Hiwa, Berun A. Abdalla, Fahmi H. Kakamad

**Affiliations:** 1 Department of Scientific Affairs, Smart Health Tower, Sulaimani, Kurdistan 46000, Iraq; 2 Kurdistan Center for Gastroenterology and Hepatology, Sulaimani, Kurdistan 46000, Iraq; 3 Kscien Organization for Scientific Research, Sulaimani, Kurdistan 46000, Iraq; 4 Department of Pathology, Sulaimani Teaching Hospital, Sulaimani, Kurdistan 46000, Iraq; 5 College of Medicine, University of Sulaimani, Sulaimani, Kurdistan 46000, Iraq

**Keywords:** traumatic neuroma, amputation neuroma, cholecystectomy, biliary stricture, cholangiocarcinoma, benign tumor

## Abstract

Laparoscopic cholecystectomy has been found to be associated with the development of traumatic neuromas on rare occasions. The present study reports a rare case of post-cholecystectomy biliary tree traumatic neuroma. Herein, a 47-year-old female with a history of laparoscopic cholecystectomy presented with upper abdominal pain and anorexia. Upon an examination, a yellow discoloration of the sclera was observed. Magnetic resonance cholangiopancreatography revealed a dilated proximal bile duct and mild dilatation of the intrahepatic biliary tree due to a stricture. Intraoperatively, a hard bile duct mass was observed with multiple enlarged lymph nodes in the peri-hepatic region. The patient was initially suspected to have bile duct cancer; however, a histopathological analysis of the resected mass revealed a bile duct traumatic neuroma. Biliary traumatic neuromas may be underestimated since they often remain asymptomatic. It is unfortunate that, as traumatic neuromas often lack distinguishing characteristics, no particular radiological findings for traumatic neuromas of the bile duct have been described to date, at least to the best of our knowledge. The rarity of this condition, combined with the absence of a standardized diagnostic modality, renders its diagnosis difficult and can even lead to misdiagnosis as biliary cancer.

## Introduction

Traumatic neuroma is a rare, non-neoplastic lesion that forms at the proximal end of a damaged nerve following trauma or surgery, as a healing process involving the hyperplastic proliferation of nerve fibers and connective tissue (1). Although it is a common pathology following trauma and surgeries, it is rarely reported in clinical practice (2). The lesion can develop after any surgery, particularly an amputation (3). The lower extremities are the most frequent site of occurrence, followed by the head and neck, radial nerve and brachial plexus (1). A biliary neuroma is a relatively rare benign tumor that can be classified into two types: Primary and traumatic neuromas (4). On rare occasions, cholecystectomy has been found to be associated with the etiology of this condition. The traumatic neuroma may mimic malignant tumors due to its vague clinical and imaging features (3). Pathologies involving the biliary tree, such as infections, inflammatory diseases, trauma, stones, or surgery, can be considered as etiological factors for traumatic neuroma (5).

The present study reports a rare case of post-laparoscopic cholecystectomy biliary tree traumatic neuroma with reactive lymph nodes that was considered to be malignant intraoperatively.

## Case report

### Patient information

A 47-year-old female patient presented to the GIT Department at Smart Health Tower, with a history of laparoscopic cholecystectomy for symptomatic gallstones, who had been affected by upper abdominal pain and anorexia 4 weeks prior to presentation. There was no vomiting or diarrhea. She had a negative past medical history.

### Clinical findings

Upon a physical examination, a yellow discoloration of the sclera without pallor was observed. Upon palpation, the abdomen was soft, non-distended and non-tender, and there was no evidence of organomegaly. Bowel sounds were positive. A digital rectal examination revealed normal-colored stool. The patient's vital signs were normal.

### Diagnostic assessment

**Figure 1:**
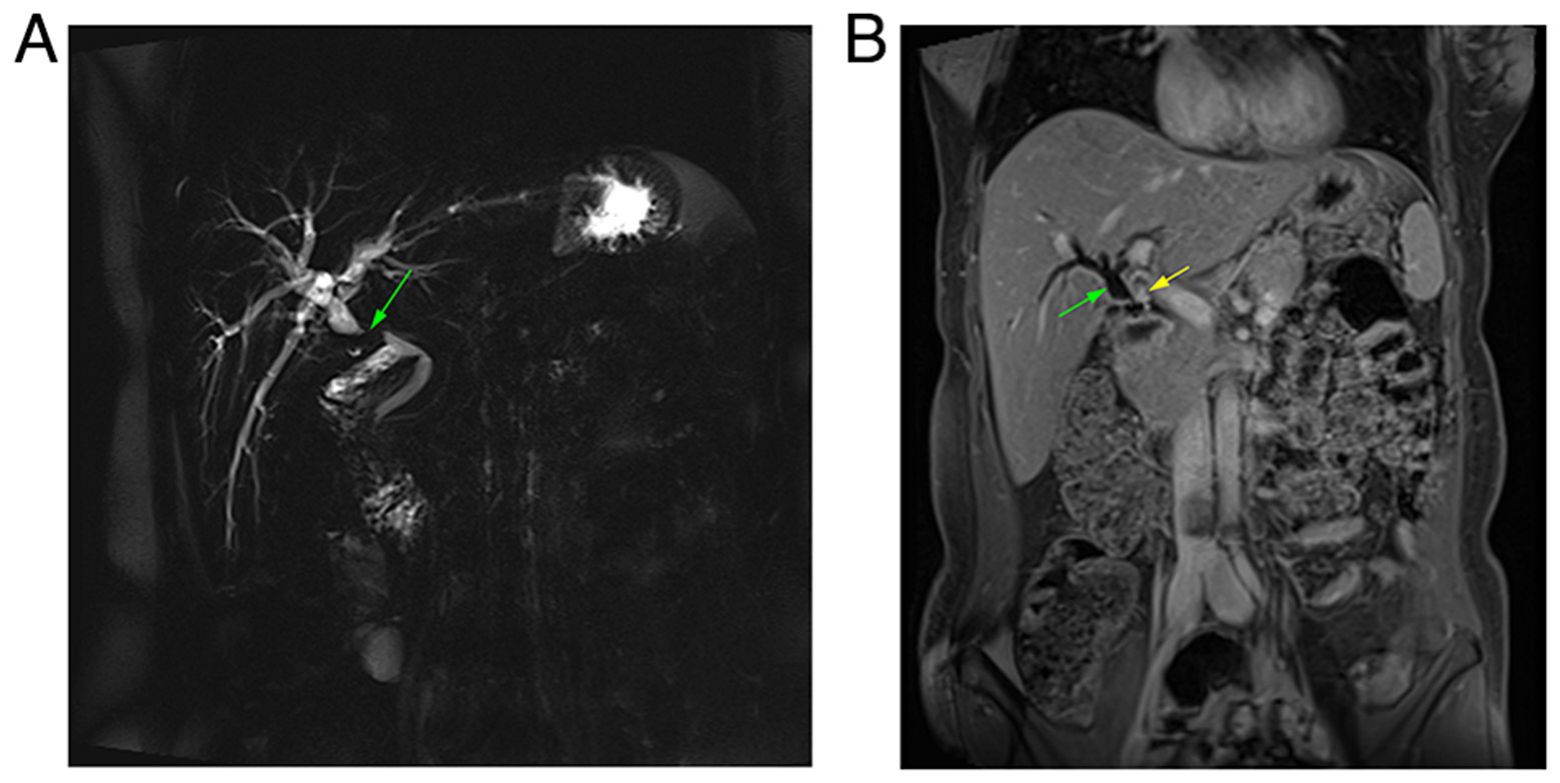
(A) Magnetic resonance cholangiopancreatography thick slab illustrating intrahepatic biliary dilatation with upper common bile duct stricture (green arrow). (B) Abdominal magnetic resonance imaging with intravenous contrast coronal section, illustrating biliary dilatation (green arrow), and a small enhanced lesion at the site of biliary obstruction (yellow arrow).

The analyses of blood parameters, including white blood cell count, serum creatinine, blood urea, C-reactive protein, serum amylase, carcinoembryonic antigen and CA 19-9 levels, revealed results within the normal range. The liver function profile was elevated (total serum bilirubin was 4.2 mg/dl and alkaline phosphatase was 250 IU/l). Magnetic resonance cholangiopancreatography (MRCP) revealed a dilated proximal bile duct and mild dilatation of the intrahepatic biliary tree due to a 1-cm stricture, 16 mm distal to the confluence of the right and left hepatic ducts (Fig. 1). Dynamic liver magnetic resonance imaging (MRI) was performed to exclude tumors, and it revealed the same finding of bile duct stricture due to a clip on the bile duct. Based on this finding, the case was diagnosed as a post-operative bile duct stricture. An endoscopic retrograde cholangiopancreatography (ERCP) was initially planned to be conducted.

### Therapeutic intervention

**Figure 2:**
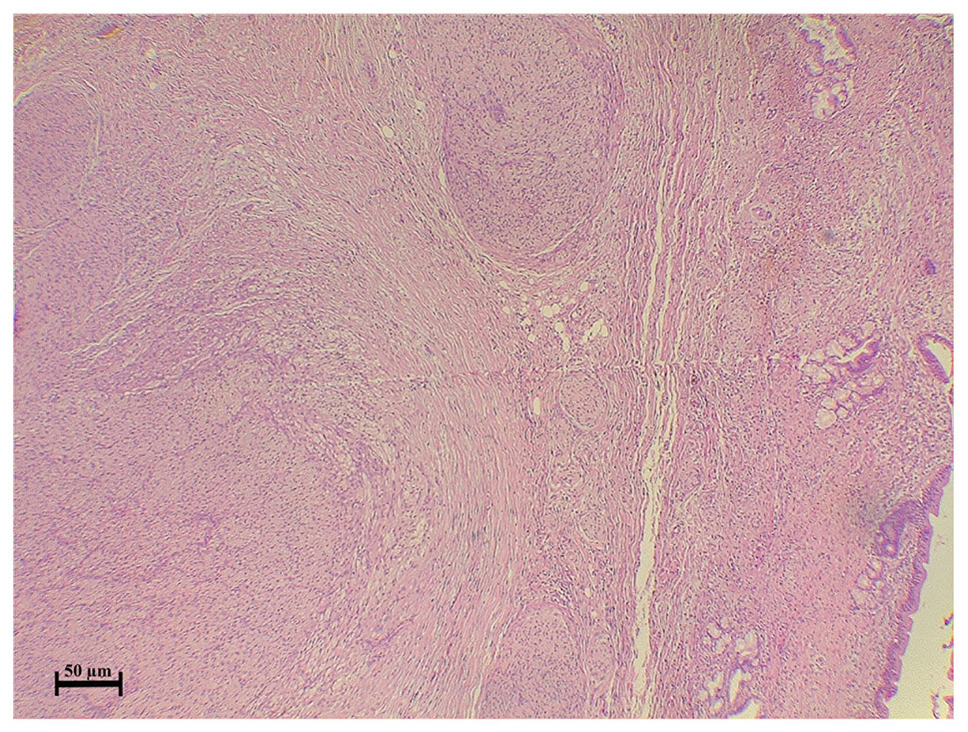
Section illustrating a common bile duct with benign epithelial lining and benign glands with wall thickening, inflammation, fibrosis and numerous disordered nerve bundles, both intra and extramurally.

**Figure 3:**
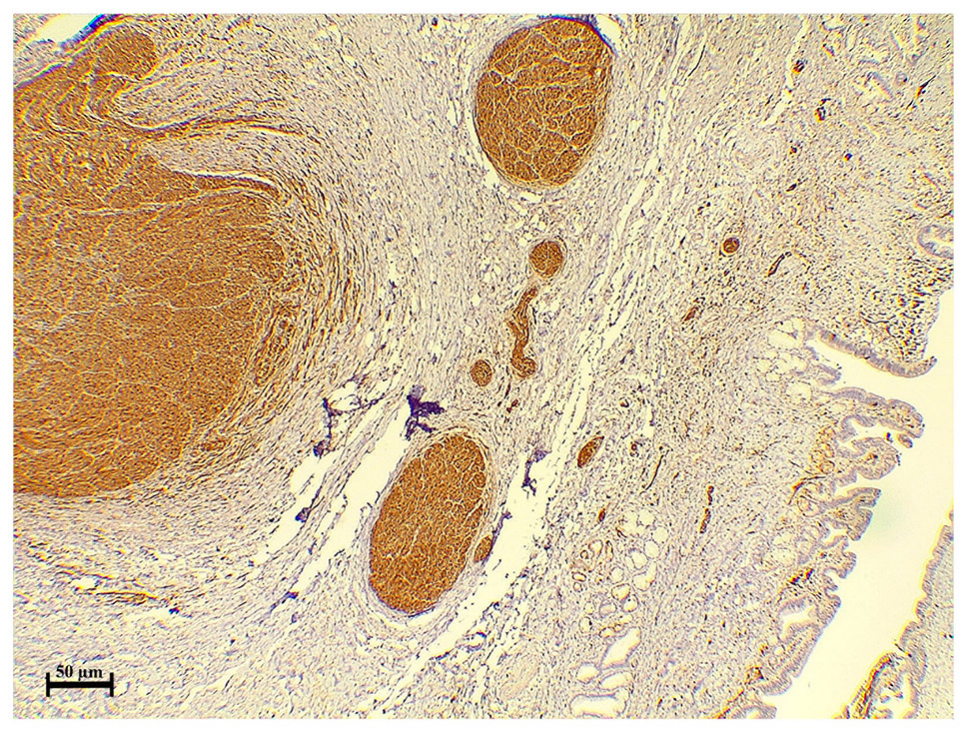
SOX10 immunostaining illustrating diffuse and strong positivity in the disorganized nerve bundles.

The case was discussed by the multidisciplinary team of Smart Health Tower, and by reviewing the imaging findings, the team suspected thickening in the area of the stricture; therefore, it was decided that surgery should be performed and not an ERCP. Under general anesthesia, a laparotomy was made through donor incisions. Intraoperatively, a hard bile duct mass of 2 cm in the greatest dimension was observed with multiple enlarged lymph nodes in the peri-hepatic region. It was suspected to be a case of bile duct cancer. As a result, bile duct excision with proximal and distal safe margins, as well as peri-hepatic lymphadenectomy, was performed (14 lymph nodes were resected). A Roux-en-Y jejunostomy was performed, and a drain was left in the subhepatic region. The resected specimens were sent for histopathological analysis, which revealed bile duct traumatic neuroma (Fig. 2). Immunostaining revealed diffuse and strong positivity in the disorganized nerve bundle (Fig. 3). SOX10 was used for the confirmation of the neural nature of the lesion because SOX10 shows an increased specificity for soft tissue tumors of neural crest origin (6).

Histopathological analysis and immunostaining were performed as follows: For immunohistochemistry, the paraffin blocks were cut into 4-6-µm-thick sections and transferred onto charged glass slides. Subsequently, they were placed in an oven at 60˚C overnight. Antigen retrieval was performed using the Dako PT Link (Agilent Technologies, Inc.) by boiling the sections at 100˚C for 5 to 10 min. A solution of pH 6.0 or pH 9.0 was used regarding the target antibody. The slides were then subjected to a 15-min wash with a 20 ml buffer solution (0.05 mol/l Tris/HCl, 0.15 mol/L NaCl, 0.05% Tween-20, pH 7.6) at room temperature. To facilitate the process, the slides were welled using the Dako Pen (Agilent Technologies, Inc.). Furthermore, endogenous peroxidase was blocked using 3% hydrogen peroxide. Subsequently, the primary antibodies (SOX-10: EP268, ready-to-use, LOT no. 06560006, Master Diagnostica) were applied at room temperature and left for 80 min. The secondary antibody, which was horseradish peroxidase (PolyDetector Plus Link, ready-to-use, LOT no. 0087XKD15, Bio SB PolyDetector Plus HRP, ready-to-use, LOT no. 0088PKD15, Bio SB, Inc.), was then applied, along with the chromogen (diaminobenzidine), both at room temperature for 15 min. To achieve counterstaining, hematoxylin Gill II (Leica Biosystems) was applied at room temperature for a duration of 30 sec. The slides were dried and coverslips were applied. A light microscope was used for the examination of the slides (Leica Microsystems GmbH).

### Follow-up

The post-operative recovery was smooth and uneventful. The patient was discharged on the 4th post-operative day in good health. Following 3 months of routine follow-up, the patient's condition was stable.

## Discussion

Adults can develop extra-hepatic bile duct strictures as a result of malignant or benign biliary tumors (4). Invasive adenocarcinoma is the most frequent cause of primary biliary tumors, while benign tumors, such as adenomas and papillomas are significantly less common, accounting for only 6% of all biliary tumors (4,7). Biliary tree traumatic neuroma is a rare benign, poorly defined, non-encapsulated lesion with an unregulated proliferation of all normal components of the nerve bundle, such as enlarged masses of axons, endoneurial cells, Schwann cells, and perineural cells in a rich collagenous matrix (8,9). The lesion is most typically observed following a radical neck dissection, orthopedic surgery or limb amputation (10,11). It is rarely reported following parotidectomy, tooth extractions, cholecystectomy and liver transplantation (12). The vast majority of these lesions in the biliary tree arise in the cystic duct remnants following cholecystectomy (13). The majority of the cases documented in the literature occurred following open cholecystectomy. Only a few cases have been recorded as a result of laparoscopic cholecystectomy (3,4). The case described herein had a history of laparoscopic cholecystectomy, which is in line with a previous report by Nechi *et al* (3).

According to several case reports and reviews, the interval between surgery and the diagnosis of biliary traumatic neuroma ranged from 2 months to 46 years, with a mean age of 5 years (4). The interval in the current case was only 4 weeks. Traumatic neuroma, often known as amputation neuroma, is exceedingly rare in the gallbladder without preceding surgery or cholelithiasis (7). Although the pathophysiology of biliary traumatic neuroma has been shown to be associated with the process of nerve regeneration, potential mechanisms encouraging its production remain unclear. Calcineurin inhibitors may be one of the elements involved in the development of biliary traumatic neuroma. Calcineurin inhibitors, in general, aid in nerve repair by promoting axon development. In addition, tacrolimus acts as a neuroprotectant and neurotrophic drug, enhancing neurological recovery following peripheral nerve and spinal cord injuries (14).

These tumors are frequently identified by accident. The tumor site may determine the patient's symptoms, which may include upper abdominal pain with features of obstructive jaundice in the common bile duct, hilar neuromas, or post-cholecystectomy pain in cystic duct stump tumors (13,15). The lesion is commonly misdiagnosed as cholangiocarcinoma, and a comprehensive treatment strategy is undertaken (15). The case described herein presented with upper abdominal pain, anorexia and yellow discoloration of the sclera. This is in contrast to the study by Nechi *et al* (3) that reported only jaundice as the presentation of their case.

The most difficult aspect of the management of biliary traumatic neuroma is the pre-operative diagnosis (13). Although various imaging techniques, such as ultrasound (US), computed tomography (CT) and MRI, are useful to some extent, the diagnosis of biliary traumatic neuroma pre-operatively remains challenging (16,17). Typically, these tumors are found following the compression of the surrounding structures, leading to the misdiagnosis as hilar cholangiocarcinoma (13). Previous studies have observed that CT and MRI clearly demonstrate biliary duct dilatation without proof of a tumor-like lesion (18,19). MRCP can reveal bile duct stenosis (18). However, none of these modalities has the sensitivity and specificity required for proper pre-surgical identification of biliary traumatic neuroma, and none can definitively rule out the existence of malignancy (13). In the case described in the present study, MRCP revealed a dilated proximal bile duct and mild dilatation of the intrahepatic biliary tree due to a stricture distal to the confluence of the right and left hepatic ducts. A dynamic liver MRI was performed to exclude tumors and revealed the same finding of bile duct stricture due to a clip on the bile duct.

Shimura *et al* (20) demonstrated that intraductal ultrasonography can differentiate traumatic neuroma from other malignancies by providing accurate details. Biopsies utilizing cholangioscopy and endoscopic US were previously used to diagnose two cases of traumatic neuroma pre-operatively (21,22).

Surgery is recommended to confirm the diagnosis and treat biliary obstruction. This is performed by resecting the extrahepatic biliary system that is closely attached to the neuroma, followed by a Roux-en-Y hepaticojejunostomy (9). The clinical manifestations in the patient described herein and the intraoperative results all suggested malignancy. Therefore, an extensive surgical approach was performed.

In conclusion, biliary tree traumatic neuroma is an uncommon occurrence following laparoscopic cholecystectomy. The rarity of this condition, combined with the absence of a standardized diagnostic modality, renders its diagnosis difficult and can even lead to its misdiagnosis as biliary cancer.

## Data Availability

The datasets used and/or analyzed during the current study are available from the corresponding author upon reasonable request.
